# Repetitive sequences: the hidden diversity of heterochromatin in prochilodontid fish

**DOI:** 10.3897/CompCytogen.v9i4.5299

**Published:** 2015-08-07

**Authors:** Maria L. Terencio, Carlos H. Schneider, Maria C. Gross, Edson Junior do Carmo, Viviane Nogaroto, Mara Cristina de Almeida, Roberto Ferreira Artoni, Marcelo R. Vicari, Eliana Feldberg

**Affiliations:** 1 Federal University of Integration American-Latin (Universidade Federal da Integração Latino-Americana), Laboratory of Genetics, Av. Tarquínio Joslin dos Santos, 1000, Jardim Universitário, Foz do Iguaçu, PR, Brazil 85857-190; 2 Federal University of Amazonas (Universidade Federal do Amazonas), Institute of Biological Sciences, Department of Genetics, Laboratory of Animal Cytogenomics, Manaus, AM, Brazil; 3 Federal University of Amazonas, Institute of Biological Sciences, Laboratory of DNA Technologies, Manaus, AM, Brazil; 4 State University of Ponta Grossa, Department of Structural and Molecular Biology and Genetics, Laboratory of Cytogenetics and Evolution, Ponta Grossa, PR, Brazil; 5 National Institute of Amazonian Research, Laboratory of Animal Genetics, Av. André Araújo, 2936, Petrópolis, Manaus, AM, Brazil 69011-970

**Keywords:** Chromosomal painting, Fish, microsatellites, repetitive sequences, sex chromosome, transposable elements

## Abstract

The structure and organization of repetitive elements in fish genomes are still relatively poorly understood, although most of these elements are believed to be located in heterochromatic regions. Repetitive elements are considered essential in evolutionary processes as hotspots for mutations and chromosomal rearrangements, among other functions – thus providing new genomic alternatives and regulatory sites for gene expression. The present study sought to characterize repetitive DNA sequences in the genomes of *Semaprochilodus insignis* (Jardine & Schomburgk, 1841) and *Semaprochilodus taeniurus* (Valenciennes, 1817) and identify regions of conserved syntenic blocks in this genome fraction of three species of Prochilodontidae (*Semaprochilodus insignis*, *Semaprochilodus taeniurus*, and *Prochilodus lineatus* (Valenciennes, 1836) by cross-FISH using *Cot-1* DNA (renaturation kinetics) probes. We found that the repetitive fractions of the genomes of *Semaprochilodus insignis* and *Semaprochilodus taeniurus* have significant amounts of conserved syntenic blocks in hybridization sites, but with low degrees of similarity between them and the genome of *Prochilodus lineatus*, especially in relation to B chromosomes. The cloning and sequencing of the repetitive genomic elements of *Semaprochilodus insignis* and *Semaprochilodus taeniurus* using *Cot-1* DNA identified 48 fragments that displayed high similarity with repetitive sequences deposited in public DNA databases and classified as microsatellites, transposons, and retrotransposons. The repetitive fractions of the *Semaprochilodus insignis* and *Semaprochilodus taeniurus* genomes exhibited high degrees of conserved syntenic blocks in terms of both the structures and locations of hybridization sites, but a low degree of similarity with the syntenic blocks of the *Prochilodus lineatus* genome. Future comparative analyses of other prochilodontidae species will be needed to advance our understanding of the organization and evolution of the genomes in this group of fish.

## Introduction

Multiple copies of DNA sequences, known as “repetitive DNA”, compose large portions of eukaryotic genomes. Repetitive DNA is generally divided into two groups: (1) tandem repeats, which include DNA satellites, minisatellites, and microsatellites; and (2) dispersed interspersed repeats composed of transposable elements (TEs) (Timberlake 1978, Charlesworth 1994, Jurka 2005), but there are other gene families with sequence repetitions also known as repetitive DNA, such as the genes encoding for ribosomal RNA (rRNA) (Long 1980). While the structure and organization of this genome fraction is still poorly understood in fish, most of these non-coding repetitive sequences appear to be located in heterochromatic regions (Fishcher et al. 2004, Martins et al. 2011).

The repetitive sequences were largely considered to be “junk”, “selfish”, or “parasitic” DNA (Doolittle and Sapienza 1980, Orgel and Crick 1980, Nowak 1994) due to the lack of any known functions in the genome for these sequences. With ever-increasing volumes of genomic information, however, these repetitive sequences are now known to play larger roles in the structural and functional evolution of the genome (Shapiro and Vonsternberg 2005, Biémont and Vieira 2006). Indeed, repetitive sequences are now known to be involved in chromosomal rearrangements and responsible for significant proportions of the karyotypic variations observed in many groups (Kidwell 2002, Schneider et al. 2013).

In Prochilodontidae, centromeric heterochromatin regions have been observed in all 54 chromosomes in all of the species analyzed, as well as in the B chromosomes of *Prochilodus lineatus* (Valenciennes, 1836) (Pauls and Bertollo 1980, Oliveira et al. 2003, Hatanaka et al. 2002, Terencio et al. 2012). However, *Semaprochilodus insignis* (Jardine & Schomburgk, 1841) has additional heterochromatic blocks in the terminal regions of the first metacentric pair, while *Semaprochilodus taeniurus* (Valenciennes, 1817) has large bitelomeric markings in metacentric pairs 2 and 3. The ZZ/ZW sex chromosome system may have originated through an *in cis* process of heterochromatin accumulation that differentiated into the W chromosome – with consequent recombination restrictions starting with the first chromosome pair (Terencio et al. 2012a).

The phylogenetic biogeography of the Prochilodontidae indicates that the family dates back minimally to approximately 12 million years ago, with higher level intrafamilial cladogenic events also dating to at least that time period; these dates are congruent with data from the fossil record for more encompassing groups within the Characiformes (Sivasundar and Bermingham 2001, Castro and Vari 2004). Phylogenies constructed based on morphological (information from osteological and soft anatomical systems) and molecular (ATPase, D-loop, ND4 and COI) characters demonstrates that *Prochilodus* is the sister group to the clade formed by *Ichthyoelephas* plus *Semaprochilodus* (Turner et al. 2004). It is believed that heterochromatic regions play important roles in the differentiation of this fish group, despite the relatively stable karyotypic macrostructures of the Prochilodontidae. The genetic composition of these regions is still only poorly understood, however, and the only firm information available concerns the presence of large amounts of repetitive DNA sequences in the B chromosomes of *Prochilodus lineatus* (Camacho and Beukeboom 2000) and in the W sex chromosome of *Semaprochilodus taeniurus* (Terencio et al. 2012b). These sequences were identified and classified as microsatellites, transposons, and retrotransposons in the latter species.

Heterochromatic regions are essential to evolutionary processes because of their ability to propagate and influence genes (Grewal and Jia 2007), and the present study therefore sought to characterize the moderate to highly repetitive DNA sequences in *Semaprochilodus insignis* and *Semaprochilodus taeniurus* by cloning and sequencing them and identifying conserved syntenic blocks of this fraction in three species of the family Prochilodontidae (*Semaprochilodus insignis*, *Semaprochilodus taeniurus*, and *Prochilodus lineatus*) using cross-FISH techniques with *Cot*-1 DNA probes.

## Materials and methods

Ten specimens of *Semaprochilodus insignis* (six females and four males) and 12 *Semaprochilodus taeniurus* (seven females and five males) were examined cytogenetically. These fish were captured with the authorization of ICMBio SISBIO 10609-1/2007 at the confluence of the Negro and Solimões Rivers (AM) and at the Amazonas and Tapajós (PA) Rivers. Five *Prochilodus lineatus* (two females and three males) were captured from the Tibagi River (PR). The fish were anesthetized in ice-cold water and were sacrificed. Voucher specimens were deposited in the INPA Animal Genetics Laboratory fish collection (10034, 10037, 10047 and 10696). Chromosome preparations were obtained from anterior kidney cells using an *in vivo* colchicine treatment (Bertollo et al. 1978). Institutional abbrevoations: UFAM, Federal University of Amazonas; INPA, National Institute of Amazonian Research; UEPG, State University of Ponta Grossa.

### Isolation of repetitive DNA via re-association kinetics

Enriched samples containing repetitive DNA sequences from *Semaprochilodus insignis* and *Semaprochilodus taeniurus* were constructed based on the renaturation kinetics of *Cot-1* DNA (DNA enriched for highly and moderately repetitive DNA sequences) according to the protocol described by Zwick et al. 2010) and recently adapted by Ferreira and Martins (2008). DNA samples (50 μl of 100-500 ng/μl of DNA in 0.3 M NaCl) were autoclaved (121 °C) for 5 minutes (min) to obtain fragments ranging from 100 to 2000 base pairs. Next, the DNA was denatured at 95 °C for 10 min, placed on ice for 10 seconds (s) and subsequently placed at 65 °C for 1 min for re-annealing. The samples were incubated at 37 °C for 8 min with 1 U of S1 nuclease to permit the digestion of single-stranded DNA. The repetitive portion of this DNA was recovered by freezing in liquid nitrogen, and the DNA was extracted using phenol-chloroform. The resulting DNA fragments were used as probes for fluorescence *in situ* hybridization, cloned and sequenced.

### Fluorescence *in situ* hybridization (FISH)

The repetitive *Semaprochilodus taeniurus* and *Semaprochilodus insignis* sequence probes isolated using *Cot-1* DNA were labeled with digoxigenin-11-dUTP and biotin-16-dUTP (Dig-Nick Translation mix and Biotin-Nick Translation mix; Roche), respectively, by nick translation reactions following the manufacturer’s instructions. Two antibodies, namely, anti-digoxigenin-rhodamine and streptavidin (Life Technologies), were used for signal detection. Fluorescence *in situ* hybridization (FISH) was performed on mitotic chromosome spreads (Pinkel et al. 1986). Homologous and heterologous *in situ* fluorescent hybridizations were performed using 77% stringency (2.5 ng/µl of DNA, 50% deionized formamide, 10% dextran sulfate and 2 × SSC at 37 °C for 18 hours). The chromosomes were counterstained with DAPI (2 µg/ml) in Vectashield mounting medium (Vector).

### Microscopy/Image Processing

Hybridized chromosomes were analyzed using an Olympus BX51 epifluorescence microscope, and the images were captured with a digital camera (Olympus DP71) using the Image-Pro MC 6.3 software.

### Cloning and sequencing of repetitive sequence

One microgram of the *Cot-1* DNA products was cloned using a pMOS Blunt-ended PCR Cloning Kit (GE Healthcare), purified using the GFX PCR Purification Kit (GE Healthcare) and sequenced using the Big Dye Kit (Applied Biosystems) in an ABI 3130 genetic analyzer. Sequence alignment was performed using Clustal W (Thompson et al. 1994), which is included in the BioEdit 7.0 software program (Hall 1999). Each clone was used as a query in BLASTN (Basic Local Alignment Search Tool nucleotide) searches against the NCBI nucleotide collection (http://www.ncbi.nlm.nih.gov) and in searches against the Repbase database (Jurka et al. 2005) at the Genetic Information Research Institute (Giri) (http://www.girinst.org/repbase/) using CENSOR software (Kohany et al. 2006).

## Results

**Figure 1.**
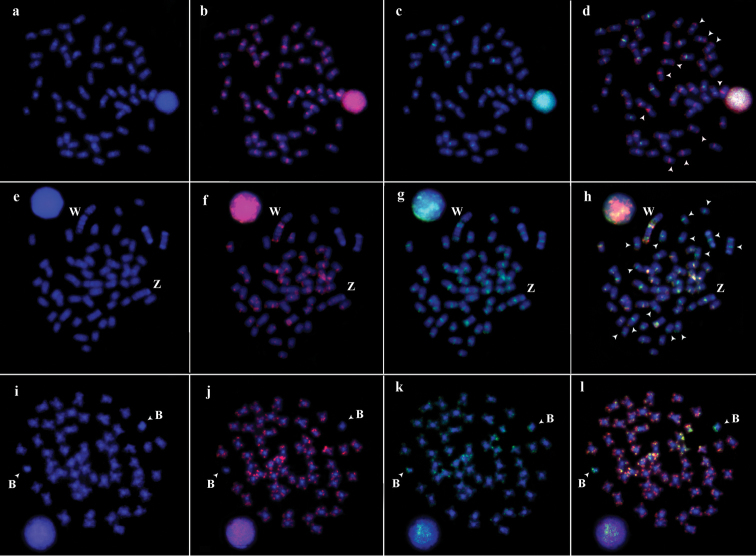
*Cot-1* DNA fraction hybridization in three species of Prochilodontidae. **a** *Semaprochilodus insignis* chromosomes counterstained with DAPI **b** *Cot-1* DNA from the *Semaprochilodus insignis* genome hybridized to its own chromosomes **c** *Cot-1* DNA from the *Semaprochilodus taeniurus* genome hybridized to *Semaprochilodus insignis* chromosomes **d** Double-FISH of the *Cot-1* DNA fraction **e** *Semaprochilodus taeniurus* chromosomes counterstained with DAPI **f** *Cot-1* DNA from the *Semaprochilodus taeniurus* genome hybridized to its own chromosomes **g** *Cot-1* DNA from the *Semaprochilodus insignis* genome hybridized to the *Semaprochilodus taeniurus* chromosomes **h** Double-FISH of the *Cot-1* DNA fraction **i** *Prochilodus lineatus* chromosomes counterstained with DAPI **j** *Cot-1* DNA from the *Semaprochilodus insiginis* genome hybridized to *Prochilodus lineatus* chromosomes **k** *Cot-1* DNA from the *Semaprochilodus taeniurus* genome hybridized to *Prochilodus lineatus* chromosomes **l** Double-FISH of the *Cot-1* DNA fraction.

Hybridization of the *Semaprochilodus insignis Cot-1* DNA probe to its own chromosomes demonstrated that the repetitive elements of its genome were located in the centromeric regions of all chromosomes, as well as in the terminal region of several chromosomes (Fig. 1a and b). The cross-hybridization of the *Semaprochilodus taeniurus Cot-1* DNA probe to the chromosomes of *Semaprochilodus insignis* revealed markers in the centromeric region, although they were smaller than those observed using species-specific probes (Fig. 1c). Additionally, no terminal markers were observed in *Semaprochilodus insignis* using the heterologous probe, indicating that this species has chromosome pairs (Fig. 1d, arrowheads) that carry species-specific repetitive sequences not shared with *Semaprochilodus taeniurus* (Fig. 1a, b, c and d).

Hybridization of the *Semaprochilodus taeniurus Cot-1* DNA probe to its own chromosomes likewise revealed that repetitive sequences were abundant in the genome of this species and located in various regions (e.g., centromeric, interstitial, and terminal) of the entire chromosome complement (Fig. 1e and g). Cross-FISH reactions were performed using the *Semaprochilodus insignis Cot-1* DNA probe and demonstrated the presence of conserved syntenic blocks in several chromosomal regions (Fig. 1f). *Semaprochilodus taeniurus* also displayed species-specific repetitive DNA sites located in the centromeric and terminal regions of 14 chromosomes (Fig. 1h, arrows), with no observed hybridizations of the *Semaprochilodus insignis Cot-1* DNA probe to these same regions (Fig. 1e, f, g, and h).

Both *Cot-1* DNA probes of *Semaprochilodus insignis* and *Semaprochilodus taeniurus* displayed positive hybridization signals in the terminal regions of the entire complement of *Prochilodus lineatus* chromosomes. The supernumerary (i.e., B) chromosomes (Fig. l, arrowheads) revealed hybridization signals only with the *Semaprochilodus taeniurus Cot-1* DNA probe. The same marker pattern seen on one of the B chromosomes was also observed on the autosomal chromosomes, while only one of the chromosome arms exhibited hybridization signals shared with the other B chromosome (Fig. 1 i, j, k and l).

Cloning and sequencing the repetitive genome elements obtained from *Semaprochilodus insignis* and *Semaprochilodus taeniurus Cot-1* DNA identified 48 DNA fragments of varying sizes (GenBank: JX848379–JX848393). 71% of repetitive DNA diversity sampled (*Cot*-1 DNA) of *Semaprochilodus insignis* displayed high similarity to microsatellites, 17% to DNA transposons, and 10% to retrotransposons (Table 1); 75% of the repetitive sequences sampled of *Semaprochilodus taeniurus* displayed high similarity to microsatellites, 5% to transposons, and 15% to retrotransposons (Table 2).

**Table 1.** Nucleotide homology of the *Cot-1* DNA fraction clones of *Semaprochilodus insignis* to known sequences in public databases. BLASTN results and their respective identities are displayed.

Isolate clone	Repetitive sequences	Similarity	Identities
**Sin1**	DNA transposon	EnSpm-3_DR (RepBase/GIRI*)	70%
**Sin2**	Microsatellite	*Cyprinus carpio* (GenBank JN756399.1)	92%
**Sin3**	Microsatellite	*Hypostomus gymnorhynchus* (GenBank HM545164.1)	83%
**Sin4**	Non-LTR retrotransposon	HERO-2_DR (RepBase/GIRI*)	78%
**Sin5**	Microsatellite/Retrotransposon	*Colossoma macropomum* (HM579956.1) SINE_2 (RepBase/GIRI*)	79%–75%
**Sin6**	DNA transposon	Mariner/Tc1 (RepBase/GIRI*)	76%
**Sin7**	DNA transposon	ERV2 Endogenous Retrovirus (RepBase/GIRI*)	77%
**Sin8**	Microsatellite	*Cyprinus carpio* (GenBank JN733372.1)	100%
**Sin9**	Non-LTR retrotransposon	Rex1 (RepBase/GIRI*)	73%
**Sin10**	Microsatellite	*Cyprinus carpio* (GenBank JN771242.1)	91%
**Sin11**	DNA transposon	*Labeo rohita*Tc1-like (GenBank AY083617.1)	77%
**Sin12**	Microsatellite	*Cyprinus carpio* (GenBank JN761177.1)	100%
**Sin13**	Microsatellite	*Hippoglossus hippoglossus* (GenBank AJ270780.1)	89%
**Sin14**	DNA transposon	Helitron-2_DR (RepBase/GIRI*)	83%
**Sin15**	Microsatellite	*Cyprinus carpio* (GenBank JN785563.1)	83%
**Sin16**	Microsatellite	*Salmo salar* CAG-repeat (GenBank Y11457.1)	87%
**Sin17**	Microsatellite	*Eleutheronema tetradactylum* (GenBank AB697177.1)	80%
**Sin18**	Microsatellite	*Oncorhynchus mykiss* (GenBank AY039630.1)	86%
**Sin20**	Microsatellite	*Prochilodus lineatus* (GenBank AY285824.1)	84%
**Sin21**	Microsatellite	*Cyprinus carpio* (GenBank JN745523.1)	89%
**Sin22**	Microsatellite	*Cyprinus carpio* (GenBank JN757227.1)	90%
**Sin23**	Microsatellite	*Cyprinus carpio* (GenBank JN737559.1)	92%
**Sin38**	Microsatellite	*Cyprinus carpio* (GenBank JN755429.1)	100%
**Sin39**	Microsatellite	*Cyprinus carpio* (GenBank JN744936.1)	92%
**Sin41**	Microsatellite	*Cyprinus carpio* (GenBank JN746351.1)	95%
**Sin42**	Microsatellite	*Cyprinus carpio* (GenBank JN757934.1)	81%
**Sin48**	Microsatellite	*Cyprinus carpio* (GenBank JN731077.1)	70%

* Database Repbase (http://www.girinst.org)

**Table 2.** Nucleotide homology of the *Cot-1* DNA fraction clones of *Semaprochilodus taeniurus* to known sequences in public databases. BLASTN results along with their respective identities are displayed.

Isolate clone	Repetitive sequences	Similarity	Identities
**Ste1**	Microsatellite	*Prochilodus mariae* (GenBank JF832400.1)	87%
**Ste2**	Microsatellite	*Epinephelus fuscoguttatus* (GenBank GU799242.1)	82%
**Ste3**	Microsatellite	*Cyprinus carpio* (GenBank JN779618.1)	96%
**Ste4**	DNA transposon	Tc1_FR2(RepBase/GIRI*)	82%
**Ste5**	Microsatellite	*Cyprinus carpio* (GenBank JN756719.1)	87%
**Ste6**	Microsatellite	*Cynoglossus semilaevis* (GenBank EU907150.1)	96%
**Ste7**	Microsatellite	*Prochilodus mariae* (GenBank JF832400.1)	84%
**Ste8**	Non-LTR retrotransposon	L2-2_DRe (RepBase/GIRI*)	86%
**Ste9**	Microsatellite	*Cyprinus carpio* (GenBank JN21488.1)	96%
**Ste10**	Microsatellite	*Cyprinus carpio* (GenBank JN731879.1)	100%
**Ste11**	Microsatellite	*Cyprinus carpio* (GenBank JN28181.1)	87%
**Ste12**	Microsatellite	*Prochilodus mariae* (GenBank JF832400.1)	80%
**Ste13**	Microsatellite	*Salmo salar* (GenBank AJ402727.1)	100%
**Ste14**	Microsatellite	*Cyprinus carpio* (GenBank JN80674.1)	95%
**Ste15**	Microsatellite	*Cyprinus carpio* (GenBank JN721488.1)	96%
**Ste16**	Adeovirus	Bovine Adenovirus type2	99%
**Ste17**	Non-LTR retrotransposon	SINE3/ 5S (RepBase/GIRI*)	82%
**Ste18**	Microsatellite	*Brycon amazonicus* (GenBank JQ993450.1)	89%
**Ste19**	Microsatellite	*Cyprinus carpio* (GenBank JN759566.1)	87%
**Ste20**	Non-LTR retrotransposon	Rex1-9_XT (RepBase/GIRI*)	75%
**Ste21**	Non-LTR retrotransposon	L2	82%

* Database Repbase (http://www.girinst.org)

## Discussion

### Diversity of repetitive DNAs in the genomes of *Semaprochilodus insignis* and *Semaprochilodus taeniurus*

Recent studies have indicated that repetitive sequences have definitely influenced genome evolution by controlling gene activity and by their involvement in chromosomal rearrangements (Valente et al. 2011). The cloning and sequencing the repetitive genome elements obtained from *Semaprochilodus insignis* and *Semaprochilodus taeniurus Cot-1* DNA displayed high similarities to repetitive sequences deposited in public DNA databases and classified as microsatellites, transposons, and retrotransposons.

Although some repetitive sequences are shared between the two *Semaprochilodus* species analyzed here, DNA sequencing indicated that the genomes of *Semaprochilodus insignis* and *Semaprochilodus taeniurus* were composed of different classes of repetitive sequences. Most of the clones displayed high similarity to microsatellites known from fish species in the order Characiformes (*Colossoma macropomum* Cuvier, 1816) and the family Prochilodontidae (*Prochilodus mariae* Eigenmann, 1922). We believed that the microsatellites were more abundant in this analysis because the method used to obtain the repetitive fraction of the genome (renaturation kinetics) generates short fragments of DNA (200−300bp) enabling the identification of microsatellites with full homology.

Microsatellites have been observed in a wide range of organisms and are common and widespread in both prokaryote and eukaryote genomes. Among the functions assigned to microsatellites are their participation in chromatin organization, DNA replication, recombination, and the regulation of gene activities (Martins et al. 2011, Li et al. 2011). In fish species such as *Steindachneridion scripta* (Miranda Ribeiro, 1918) *Rineloricaria latirostris* (Boulenger, 1900), and *Danio rerio* (Hamilton, 1822) these repetitive sequences tend to be clustered in the centromeric and telomeric regions (Shimoda et al. 1999, Vanzela et al. 2002). Future studies aimed at mapping microsatellites within the chromosomes of Prochilodontidae will further our understanding of the roles of those sequences in chromosomal evolution in that group.

A certain proportion of these DNA fragments displayed high degrees of similarity to transposable elements (i.e., both transposons and retrotransposons) (Tables 1 and 2) found in the genomes of fish species such as *Xiphophorus maculates* Gunther, 1866, *Leporinus elongatus* Valenciennes, 1850 and *Oryzias hubbsi* Roberts, 1998 (Volff et al. 2000, Bohne et al. 2012, Marreta et al. 2012, Takehana et al. 2012).

The sequences described in the present study may play an evolutionary role in the genomes of *Semaprochilodus insignis* and *Semaprochilodus taeniurus* as one of the sequences identified in the genome of *Semaprochilodus taeniurus* (Ste17) displayed 82% homology with a retrotransposon called SINE3 identified by (Kapitonov and Jurka 2003) as originating from 5S rRNA. As other species of the Prochilodontidae family have only one pair of chromosomes carrying these ribosomal sites, this information strengthens the hypothesis that the multiple 5S rDNA sites observed in *Semaprochilodus insignis* and *Semaprochilodus taeniurus* are pseudogenes (or repetitive sequences) derived from 5S rDNA (Terencio et al. 2012a). In *Gymnotus paraguensis* (Albert & Crampton, 2003) the multiplication of 5S rDNA gene clusters might has be caused by the involvement of transposable elements because the NTS has high identity (90%) with a Tc1-like transposon (Silva et al. 2011).

We were also able to identify sequences in *Semaprochilodus insignis* that exhibited high similarity with the transposable element Helitron. In maize, this TE seems to continually produce new non autonomous elements responsible for the duplicative insertion of gene segments at new locations and for the unprecedented genomic diversity of this species (Morgante et al. 2005). Intact Helitron elements were identified in the sex-determining region of the sex chromosomes of the platyfish *Xiphophorus maculatus*, suggesting that TE are still active in the genome of platyfish and related species – where they may have roles in the evolution of sex chromosomes and other genomic regions (Zhou et al. 2003).

*Tc1/mariner* (isolated from the genomes of *Semaprochilodus insignis* and *Semaprochilodus taeniurus*) are the most widespread superfamily of DNA transposons and can be found in fungi, plants, ciliates, and animals (including nematodes, arthropods, fish, frogs, and humans). Most of the transposon copies isolated from vertebrates are clearly inactive remnants of once active transposons that were inactivated by mutations, but only after successfully colonizing their genomes (Plasterk et al. 2009, Ivics et al. 2006).

The retroelement Rex1 was also detected in the repetitive fraction of the genomes of *Semaprochilodus insignis* and *Semaprochilodus taeniurus*. The Rex family has been widely studied in fish, and a number of different lineages have been described in this group (Volff et al. 2000) where they are known to be scattered or grouped into conspicuous clusters in the chromosomes of Neotropical cichlids (Mazzuchelli and Martins 2009, Gross et al. 2009, Teixeira et al. 2009, Oliveira et al. 1999). These elements display compartmentalized distributions in some autosomes and show clear signals along the full lengths of W chromosomes in *Semaprochilodus taeniurus* (Terencio et al. 2012).

The Line2 element was detected only in the repetitive fraction of the *Semaprochilodus taeniurus* genome. This repetitive sequence may be present in the *Semaprochilodus insignis* genome but simply not sampled in our study, or alternatively, it may have been eliminated from the genome of this species. FISH showed that Line2 sequences are organized in small clusters dispersed over all of the chromosomes of *Oreochromis niloticus* (Linnaeus, 1758), but with higher concentrations near chromosome ends (Oliveira et al. 1999). Line elements in mammals appear clustered in the G-banding regions of the chromosomes, and on the sex chromosomes in some cases (Wichman et al. 1992, Fishcher et al. 2004).

### Repetitive DNA organization in chromosomes

Repetitive DNA sequences comprising mostly the heterochromatic portions of the genome were observed using the C-banding technique. Previous studies (Feldberg et al. 1987, Vicari et al. 2006, Terencio et al. 2012b) revealed that this technique revealed that repetitive DNA sequence fractions in the genomes of fish of the family Prochilodontidae are not abundant and are located mainly in the centromeric regions of the chromosomes and, less frequently, in the terminal regions of the long arms of some chromosome pairs. Large heterochromatic blocks can be observed, however, in the supernumerary chromosomes (i.e., the B chromosomes) of *Prochilodus* spp. (Vicari et al. 2006, Voltolin et al. 2011) and in the W sex chromosome of *Semaprochilodus taeniurus* (Feldberg et al. 1987, Terencio et al. 2012a). Fluorescence *in situ* hybridization using species-specific probes of the repetitive fractions of the genomes partially confirmed the heterochromatic pattern demonstrated with the C-banding technique in both *Semaprochilodus insignis* and *Semaprochilodus taeniurus* – and positive signals were detected in the centromeric regions of all of their chromosomes. Markers were also observed in the terminal regions of some chromosomes, confirming that repetitive DNAs are also present in this area, although heterochromatin was not observed. Repetitive sequences located outside of heterochromatic regions are believed to significantly influence genome evolution, particularly by controlling and regulating gene activities, and genome sequencing has frequently revealed short and truncated copies of repetitive sequences in euchromatic genomic regions (Fischer et al. 2002, Biémont and Vieira 2006, Timberlake 1978, Yuan and Wessler 2011, Torres et al. 2011). This observation does not necessarily indicate that these repetitive sequences are constitutively expressed, however, since they tend to be silenced and undergo subsequent molecular deterioration. In other words, these sequences becomes inactive and progressively accumulate mutations, insertions, and deletions at neutral rates until completely losing their identities or become lost in the host genome (Fernández-Medina et al. 2012). The presence of repetitive DNAs in euchromatic regions has been observed in many groups, such as insects (Cabral-de-Mello et al. 2011), fish (Teixeira et al. 2009, Valente et al. 2011), and lizards (Pokorná et al. 2011), and these TEs have acquired structural/regulatory functions so that their accumulation in euchromatic regions may lend advantages to the host genome.

Cross-hybridizations of *Semaprochilodus insignis* and *Semaprochilodus taeniurus* showed patterns similar to those observed in homologous hybridization – which suggests that this portion of the genome has been conserved throughout evolution, perhaps due to a functional role. However, revealed that these species have species-specific centromeric and terminal sites not identified by heterologous hybridization.

Cross-FISH using *Semaprochilodus insignis* and *Semaprochilodus taeniurus Cot-1* DNA probes revealed hybridization signals in the subterminal regions of *Prochilodus lineatus*, in contrast to the heterochromatic pattern revealed by the C-banding technique with heterochromatin blocks being primarily observed in the centromeric region (Pauls and Bertollo 1990, Venere et al. 1990, Cavallaro et al. 2000, Artoni et al. 2006, Vicari et al. 2006, Voltolin et al. 2011). These data indicate that the repetitive fraction of centromeric heterochromatin of *Prochilodus lineatus* is different from the other two species examined (*Semaprochilodus insignis* and *Semaprochilodus taeniurus*) and that shared repetitive sequences are located on the subtelomeric portions of their chromosomes. This same pattern was observed in three species of *Prochilodus* using (AATTT)n microsatellite (Hatanaka et al. 2002) and W-specific probes (Terencio et al. 2012b).

A common karyotypic feature of species belonging to the genus *Prochilodus* is the presence of supernumerary chromosomes (B chromosomes). Many studies of B chromosomes have indicated that these supernumerary chromosomes are rich in repetitive sequences and, in certain cases, may contain a number of functional genes (Camacho 2005, Ruiz-Estévez et al. 2012). The *Prochilodus lineatus* population analyzed in the present study displayed two B chromosomes with distinct hybridization sites. The *Semaprochilodus insignis Cot-1* DNA probe did not hybridize to the B chromosomes, possibly because the repetitive fraction of the *Semaprochilodus insignis* genome is not shared with the B chromosomes of *Prochilodus lineatus*. One possible hypothesis explaining this result would be that these repetitive sequences have undergone rapid differentiation and evolution in the genome of *Semaprochilodus insignis*, resulting in a loss of homology with sequences on the B chromosomes of *Prochilodus lineatus*. Another explanation could be due to the fact it is different genera, and therefore the Bs found in each species could have different origins. The *Semaprochilodus taeniurus Cot-1* DNA probe was positive, revealing sequence sharing with *Prochilodus lineatus* B chromosomes. A number of studies have suggested that B chromosomes can influence sex determination in fish (Noleto et al. 2012, Yoshida et al. 2012), although no relationship between the occurrence of B chromosomes and sex determination has been observed in *Prochilodus lineatus*. The B chromosomes detected in *Prochilodus lineatus* were recently shown to demonstrate positive signals when hybridized with the W-specific probe, indicating the sharing of repetitive DNA families between these two species (Terencio et al. 2012a).

## Conclusions

Results from DNA sequencing indicated that the genomes of *Semaprochilodus insignis* and *Semaprochilodus taeniurus* comprise different classes of repetitive sequences that may have played important roles in their evolution. The repetitive fractions of the *Semaprochilodus insignis* and *Semaprochilodus taeniurus* genomes also exhibit high degrees of conserved syntenic blocks in terms of both the structure and location of hybridization sites. However, the genomes of both *Semaprochilodus insignis* and *Semaprochilodus taeniurus* displayed a low degree of syntenic blocks with the *Prochilodus lineatus* genome, especially with regard to the B chromosome, and the origin of this situation has not yet been elucidated.

## Authors’ contributions

MLT, CHS and MCG collected the samples, collaborated on all cytogenetic procedures, undertook the bibliographic review and coordinated the writing of the manuscript. EJC, VN, RFA, MCA and MRV participated in the development of the laboratory techniques, performed the specific W-probe for *Semaprochilodus* and reviewed the manuscript. EF coordinated the study and reviewed the manuscript. All authors read and approved of the final manuscript.
